# Beta Value Coupled Wave Theory for Nonslanted Reflection Gratings

**DOI:** 10.1155/2014/513734

**Published:** 2014-02-26

**Authors:** Cristian Neipp, Jorge Francés, Sergi Gallego, Sergio Bleda, Francisco Javier Martínez, Inmaculada Pascual, Augusto Beléndez

**Affiliations:** ^1^Departamento de Física, Ingeniería de Sistemas y Teoría de la Señal, Universidad de Alicante, Apartado de Correos 99, 03080 Alicante, Spain; ^2^Instituto Universitario de Física Aplicada a las Ciencias y las Tecnologías, Universidad de Alicante, Apartado de Correos 99, 03080 Alicante, Spain; ^3^Departamento de Óptica, Farmacología y Anatomía, Universidad de Alicante, Apartado de Correos 99, 03080 Alicante, Spain

## Abstract

We present a modified coupled wave theory to describe the properties of nonslanted reflection volume diffraction gratings. The method is based on the beta value coupled wave theory, which will be corrected by using appropriate boundary conditions. The use of this correction allows predicting the efficiency of the reflected order for nonslanted reflection gratings embedded in two media with different refractive indices. The results obtained by using this method will be compared to those obtained using a matrix method, which gives exact solutions in terms of Mathieu functions, and also to Kogelnik's coupled wave theory. As will be demonstrated, the technique presented in this paper means a significant improvement over Kogelnik's coupled wave theory.

## 1. Introduction

Kogelnik's coupled wave theory is one of the most popular theories used to calculate the efficiency of the orders that propagate inside a volume diffraction grating [1]. This is mainly due to its simplicity and the fact that analytical expressions can be obtained. This theory predicts the efficiencies of the zero and first diffracted orders for volume phase gratings, and since its introduction it has been widely used by the optics community to extract information on the properties of the recording materials. Nonetheless, the prediction capacity of Kogelnik's theory is limited in some cases, so rigorous theories such as the rigorous coupled wave (RCW) theory [2] which does not disregard the second derivatives in the coupled wave equations are needed. Since its first introduction by Moharam and Gaylord [2], the RCW method has accomplished the task of modelling different periodic structures [3–10] with precision and is also used to test the validity of more approximated theories. Although exact predictions can be obtained by using the RCW method, it is still interesting to work with analytical expressions, as they provide a clearer interpretation of the influence of the different medium parameters on the efficiency of the different orders.

One of the assumptions of Kogelnik's theory is that the grating is index-matched. That is, it is embedded in two media with the same refractive index as the average refractive index of the sinusoidal grating. Otherwise, the diffraction efficiencies must be corrected by using Fresnel expressions to account for losses at the interfaces of the different media. As demonstrated by different authors, these corrections deviate from the rigorous results, and, as cited by Wu and Glytsis [11], the errors could be as high as 30%. This fact is critical for nonslanted reflection gratings where the reflected diffracted order interacts with the specularly reflected wave at the interface air-grating. One of the strategies to account for these boundary effects is to include in the model multiple reflections [12], but there are still slight deviations from the exact results due to the effects of boundary diffraction [13–15]. In this paper, we will assume a more general form of the boundary conditions to avoid these difficulties, following a similar strategy as that proposed by Sheridan and Solymar [13] to take into account the effects of boundary diffraction.

Another disadvantage of Kogelnik's theory is that for off-Bragg incidence the direction of the diffracted order is not correctly predicted as has been pointed out by Fally et al. [16, 17]. This is due to the fact that the modulus of the propagation vector of the diffracted beam at off-Bragg incidence deviates from the expected value. A natural correction to Kogelnik's coupled wave theory to account for the last fact was made by Uchida [18] using the so-called beta value technique. As demonstrated by Fally et al., this correction predicts the experimentally observed direction of the diffracted beam. Moreover, in this paper, we will demonstrate how this beta value coupled wave theory also predicts with accuracy the diffraction efficiency for off-Bragg angles, whereas Kogelnik's theory does not.

The paper is organized as follows. In Section 2, the method is introduced, solving the beta value coupled differential equations with adequate boundary conditions. The results obtained will be compared with those obtained by using Kogelnik's coupled wave theory and a matrix method which gives exact solutions in terms of Mathieu functions. In Sections 3, 4, and 5, the general solution is particularized for the cases of a homogeneous dielectric slab (no index modulation), an index-matched dielectric grating, and a partially index-matched dielectric grating, respectively. As will be shown, the use of the beta value coupled equations combined with appropriate boundary conditions is the correct strategy if a first-order coupled wave theory is to be used for the simulation of volume reflection gratings.

## 2. Solution of the Coupled Wave Equations: General Solution

**Figure 1:**
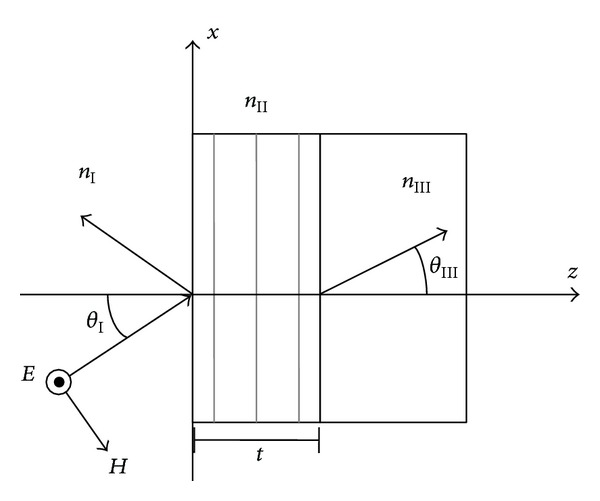
Diffraction reflection grating.

We are going to solve the problem of a nonslanted reflection grating of thickness *t*, embedded in two different media with refractive indexes *n*_I_, *n*_III_ (see Figure 1). The study will be performed for TE polarization.

The refractive index inside the grating is assumed to vary in the form:

(1) $$\begin{array}{c} n=n_{\text{II}}+\Delta n\cos\left(Kz\right), \end{array}$$

where *n*_II_ is the average refractive index, Δ*n* is the refractive index modulation, and *K* is the modulus of the grating vector, which is related to the grating period, Λ, through *K* = 2*π*/Λ.

In medium I, we will assume an incident wave of unit amplitude and a reflected wave given, respectively, by the following expressions:

(2) $$\begin{array}{c} \exp\left[-j\left(k_{x}^{\text{I}}x+k_{z}^{\text{I}}z\right)\right],\\ R\exp\left[-j\left(k_{x}^{\text{I}}x-k_{z}^{\text{I}}z\right)\right]. \end{array}$$

In medium II, the reflection grating, we will assume the existence of the zero-order diffracted wave and the first-order diffractive wave given, respectively, by the following:

(3) $$\begin{array}{c} E_{0}\left(z\right)\exp\left[-j\left(k_{x}^{\text{II}}x+k_{z}^{\text{II}}z\right)\right],\\ E_{1}\left(z\right)\exp\left[-j\left(k_{x}^{\text{II}}x-k_{z}^{\text{II}}z\right)\right]. \end{array}$$

Finally, in medium III, only the existence of a transmitted wave will be supposed, admitting that no light impinges on the grating from medium III. Consider the following:

(4) $$\begin{array}{c} T\exp\left[-j\left(k_{x}^{\text{III}}x+k_{z}^{\text{III}}z\right)\right]. \end{array}$$

The modulus of the propagation vectors in the different media is given by the following:

(5) $$\begin{array}{c} k^{i}=n_{i}\beta ,\;i=\text{I},\text{II},\text{II}, \end{array}$$

where *β* = 2*π*/*λ*, *λ*, being the wavelength in vacuum.

Now, in medium II, the nonslanted reflection grating, the amplitudes of the first-order diffracted wave, *E*_1_, and the zero-order diffracted wave, *E*_0_, are assumed to be related by the beta value coupled wave equations [18]:

(6) $$\begin{array}{c} c_{R}\frac{dE_{0}}{dz}+j\kappa E_{1}\exp\left[j\phi z\right]=0,\\ -c_{R}\frac{dE_{1}}{dz}+j\kappa E_{0}\exp\left[-j\phi z\right]=0. \end{array}$$

**Figure 2:**
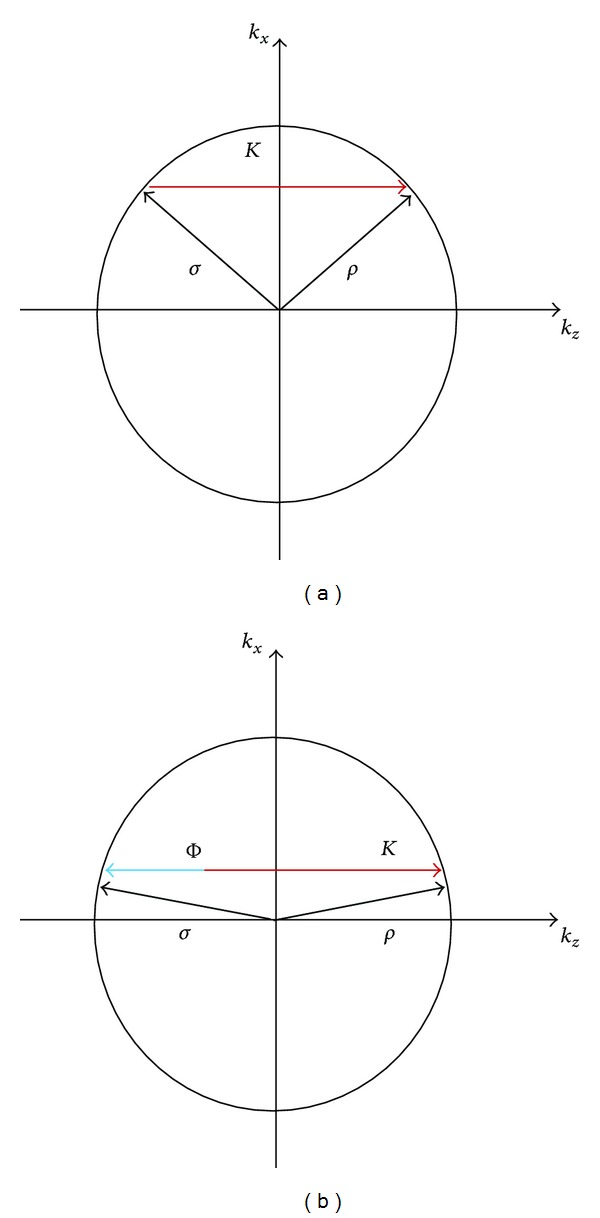
Reconstruction of a reflection diffraction grating (parameters of the *β* value model):(a) at Bragg; (b) out of Bragg.

Here, *c*_*R*_ is the cosine of the angle made by the zero-order diffracted wave with the *z*-axis, *ϕ* is the off-Bragg parameter (see Figure 2), and *κ* is the grating strength, which is related to the refractive index modulation by *κ* = *π*Δ*n*/*λ*.

Equations (6) obtained by Uchida [18] are slightly different from Kogelnik's coupled wave equations. The main difference relies on the evaluation of the off-Bragg parameter, which in the beta value model is introduced by the parameter *ϕ*. If $\vec{\rho }$ stands for the propagation vector of the zero order and $\vec{\sigma }$ refers to that of the first order, $\vec{K}$ being the grating vector, the off-Bragg parameter *ϕ* is evaluated through the following equation:

(7) $$\begin{array}{c} \vec{\sigma }=\vec{\rho }-\vec{K}\pm \phi \vec{n}, \end{array}$$

where $\vec{n}$ is a unit vector perpendicular to the interfaces (in this case in the *z* direction). The reason of (7) was to correctly account for off-Bragg incidence. In Kogelnik's theory at off-Bragg incidence, the modulus of the diffracted propagation vector is no longer *n*_II_ *β*; that is, the diffracted propagation vector lies outside the Ewald sphere, whereas through the correction introduced by Uchida [18] the propagation vector of the diffracted order is ensured to lie inside the Ewald sphere. Not only is the correct direction of the first-order diffracted beam accomplished, but also the right angular behavior of the diffraction efficiency is predicted by using the beta value technique.

For the particular case of nonslanted reflection gratings, (7) takes the form

(8) $$\begin{array}{c} \sigma_{z}=\rho_{z}-K\pm \phi . \end{array}$$

Using the notation used in this paper, we can finally relate the off-Bragg parameter to the grating vector through

(9) $$\begin{array}{c} \phi =\pm \left(2k_{z}^{\text{II}}-K\right). \end{array}$$

In general, the way of solving (6) or the coupled equations derived by Kogelnik was to impose the conditions *E*_0_(0) = 1 and *E*_1_(*t*) = 0 for reflection gratings. The expressions of the diffraction and transmission efficiency can then be obtained analytically. Nonetheless, although this case will be analysed in this work, these analytical expressions must be corrected to take into account Fresnel losses. In the particular case of nonslanted reflection gratings embedded in media with different refractive indices, this strategy is no longer valid, since the reflected diffractive beam interferes with the specularly reflected beam at the first interface. To avoid these difficulties, we will impose more general boundary conditions, which are, as will be demonstrated, the correct strategy combined with the beta value technique. To find more general boundary conditions so as to solve (6), we will use the same method as that proposed by Sheridan et al. [13–15].

Matching the electric field at *z* = 0,

(10) $$\begin{array}{c} 1+R=E_{0}\left(0\right)+E_{1}\left(0\right). \end{array}$$

Take into account that for TE polarization *H*_*x*_ is related to *E*_*y*_ through

(11) $$\begin{array}{c} H_{x}=\frac{1}{j\omega \mu }\frac{\partial E_{y}}{\partial z}. \end{array}$$

Here, *ω* is the angular frequency of the incident wave and **μ** is the magnetic permeability.

*H*_*x*_ can now be matched at the boundary at *z* = 0 giving

(12) $$\begin{array}{c} -jk_{z}^{\text{I}}\left(1-R\right)={\frac{dE_{0}}{dz}|}_{z=0}-jk_{z}^{\text{II}}E_{0}\left(0\right)+{\frac{dE_{1}}{dz}|}_{z=0}+jk_{z}^{\text{II}}E_{1}\left(0\right). \end{array}$$

Matching the electric field at *z* = *t*.

(13) $$\begin{array}{c} {Te}^{-jk_{z}^{\text{III}}t}=E_{0}\left(t\right)e^{-jk_{z}^{\text{II}}t}+E_{1}\left(t\right)e^{jk_{z}^{\text{II}}t}. \end{array}$$

And, finally, matching *H*_*x*_ at the boundary *z* = *t*,

(14) −jkzIIITe−jkzIIIt ={−jkzIIE0(t)+dE0dz|z=t}e−jkzIIt  +{jkzIIE1(t)+dE1dz|z=t}ejkzIIt.

The derivatives of the amplitudes *E*_0_ and *E*_1_ that appear in (12) and (14) can be related to the amplitudes by using (6) evaluated at *z* = 0 and *z* = *t*.

From (10) and (12), we can now obtain an appropriate boundary condition relating *E*_0_ and *E*_1_ at *z* = 0. Consider the following:

(15) $$\begin{array}{c} \alpha E_{0}\left(0\right)+bE_{1}\left(0\right)=2. \end{array}$$

In the same way, the following condition can be obtained at *z* = *t*:

(16) $$\begin{array}{c} cE_{0}\left(t\right)+dE_{1}\left(t\right)=0, \end{array}$$

where the following parameters are defined:

(17) a=1+nIIcos⁡⁡θIInIcos⁡⁡θI−κnIβcos⁡⁡θIcos⁡⁡θII,b=1−nIIcos⁡⁡θIInIcos⁡⁡θI+κnIβcos⁡⁡θIcos⁡⁡θII,c=(1−nIIcos⁡⁡θIInIcos⁡⁡θI)e−jnIIβcos⁡⁡θIIt +κnIβcos⁡⁡θIcos⁡⁡θIIe−jϕtejnIβcos⁡⁡θIIt,d=(1+nIIcos⁡⁡θIInIcos⁡⁡θI)ejnIIβcos⁡⁡θIIt −κnIβcos⁡⁡θIcos⁡⁡θIIejϕte−jnIIβcos⁡⁡θIIt.

Once the appropriate boundary conditions are obtained, the system of (6) can be solved with conditions (15) and (16).

From (10), the amplitude of the reflected wave in medium I can be obtained as

(18) $$\begin{array}{c} R=E_{0}\left(0\right)+E_{1}\left(0\right)-1. \end{array}$$

And, finally, the diffraction efficiency is calculated as

(19) $$\begin{array}{c} \eta =RR^{*}, \end{array}$$

where * indicates complex conjugate.

After solving the system of differential equations and evaluating *E*_0_(*z*) and *E*_1_(*z*) at *z* = 0, the solution of *R* through (18) can be expressed as a fraction of linear combination of exponentials:

(20) $$\begin{array}{c} R=\frac{A\left(e^{\gamma_{1}t}+e^{\gamma_{2}t}\right)+B\left(e^{\gamma_{1}t}-e^{\gamma_{2}t}\right)}{C\left(e^{\gamma_{1}t}+e^{\gamma_{2}t}\right)+D\left(e^{\gamma_{1}t}-e^{\gamma_{2}t}\right)}, \end{array}$$

where the following parameters are defined:

(21) $$\begin{array}{c} \gamma_{1,2}=\frac{1}{2}\left(j\phi \pm \sqrt{\frac{4\kappa^{2}}{c_{R}^{2}}-\phi^{2}}\right),\\ \alpha =\frac{1}{2}\sqrt{\frac{4\kappa^{2}}{c_{R}^{2}}-\phi^{2}},\\ A=2jc_{R}^{2}\alpha \left[bd-ace^{j\phi t}\right],\\ B=2c_{R}\kappa \left[ad+bce^{j\phi t}\right]-c_{R}^{2}\phi \left[bd+ace^{j\phi t}\right],\\ C=2jc_{R}^{2}\alpha \left[ad-bce^{j\phi t}\right],\\ D=2c_{R}\kappa \left[bd+ace^{j\phi t}\right]-c_{R}^{2}\phi \left[ad+bce^{j\phi t}\right]. \end{array}$$

Expression (20) can also be expressed in terms of hyperbolic functions as

(22) $$\begin{array}{c} R=\frac{A\cosh\left(\alpha t\right)+B\sinh\left(\alpha t\right)}{C\cosh\left(\alpha t\right)+D\sinh\left(\alpha t\right)}. \end{array}$$

**Figure 3:**
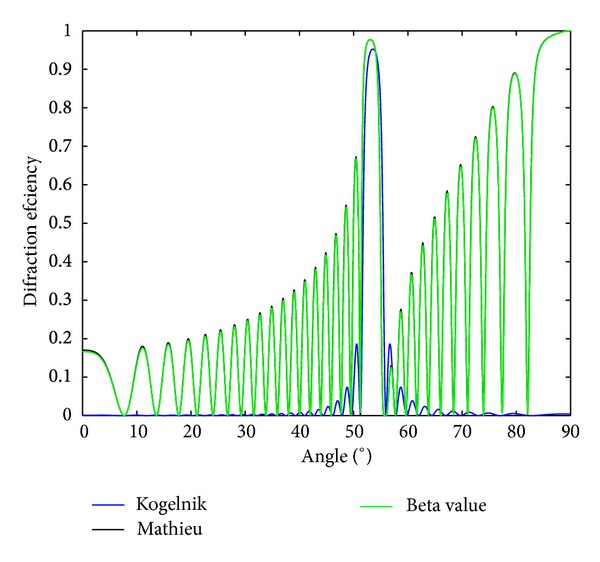
Diffraction efficiency of a reflection grating embedded in air with thickness of 25 **μ**m, spatial frequency of 4000 lines/mm, average refractive index *n*_2_ = 1.5, and refractive index modulation of 0.015.

Now, we want to check the validity of expression (22). To do this, the results obtained by using the proposed method will be compared to those obtained using a matrix method [19]. The matrix method gives exact results for the efficiency of the first diffracted order in terms of Mathieu functions. The parameters used in the simulations throughout the text, such as the refractive index, the refractive index modulation, or the thickness of the layer, were chosen to represent reflection diffraction gratings recorded in photopolymers [20–23]. In Figure 3, the diffraction efficiency as a function of the angle is represented for a nonslanted reflection grating of 4000 lines/mm, thickness of 25 **μ**m, refractive index modulation of 0.015, and average refractive index of 1.5; the grating is embedded in air. The results obtained using Kogelnik's expression are also included. As can be seen in the figure, the results obtained by using both methods are in good agreement. It is also interesting to see that Kogelnik's theory predicts with certain accuracy the position and the size of the lobe corresponding to Bragg angle, but a slight deviation of the Bragg angle is also observed.

## 3. No Index Modulation Case

This section is included for completeness, since, in general, the coupled wave theories do not give the correct response in the limiting case of no index modulation. Nonetheless, due to the particularities of this case, a correct expression can be derived from the general case when there is no index modulation.

When *κ* is equal to 0, then

(23) $$\begin{array}{c} \gamma_{1}=j\phi ,\;\;\gamma_{2}=0. \end{array}$$

So the expression of *R* is given by

(24) $$\begin{array}{c} R=\frac{\left(bd-ace^{j\phi t}\right)\left(e^{j\phi t}+1\right)+\left(bd+ace^{j\phi t}\right)\left(e^{j\phi t}-1\right)}{\left(ad-bce^{j\phi t}\right)\;\;\left(e^{j\phi t}+1\right)+\left(ad+bce^{j\phi t}\right)\left(e^{j\phi t}-1\right)}. \end{array}$$

And, finally, after some algebra,

(25) $$\begin{array}{c} R=\frac{bd-ac}{ad-bc}. \end{array}$$

If one substitutes the values of *a*, *b*, *c*, and *d* for *κ* = 0 in (25) and after little algebra, the same expression as that of the reflectance of a homogeneous dielectric slab [24] can be obtained.

It is interesting to notice that this equation could have also been obtained, by observing that if *κ* = 0 from (6) *E*_0_ and *E*_1_are constants. Consider the following:

(26) $$\begin{array}{c} E_{0}\left(z\right)=E_{0},\;\;E_{1}\left(z\right)=E_{1}. \end{array}$$

Now, from the boundary equations (15) and (16),

(27) $$\begin{array}{c} E_{0}=\frac{2d}{ad-bc},\;\;E_{1}=-\frac{2c}{ad-bc}. \end{array}$$

**Figure 4:**
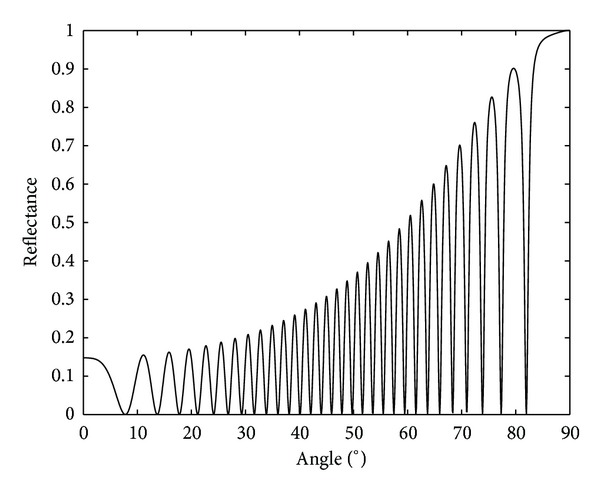
Reflectance of a homogeneous dielectric slab embedded in air with thickness 25 **μ**m and refractive index 1.5.

So, finally, using (18), the same expression as (25) can be obtained.  Figure 4 shows the reflectance as a function of the angle for a homogeneous dielectric slab of the same characteristics of the one of Figure 3, that is, with a refractive index of 1.5 and a depth of 25 **μ**m. From the comparison of both figures, it is clear why Kogelnik's theory is not applicable. That is, since the specular reflection is not included in Kogelnik's model, it cannot explain the existence of the lateral lobes with high intensity at both sides of the main lobe centered at 54° in Figure 3.

## 4. Index-Matched Case

In this case, the reflection grating is embedded in two media with the same refractive index as the average refractive index, *n*_II_, described. In this case, *a* = 2, *b* = *c* = 0, and *d* = 1. The boundary conditions are then

(28) $$\begin{array}{c} E_{0}\left(0\right)=1,\;\;E_{1}\left(t\right)=0. \end{array}$$

These conditions were used by Kogelnik to obtain the expression of the diffraction efficiency. In this particular case, the expression obtained for nonslanted reflection gratings using the beta value technique is

(29) $$\begin{array}{c} \eta =\frac{\cosh\left(2\Phi \right)-1}{1+2\Psi +\cosh\left(2\Phi \right)}, \end{array}$$

where the following parameters are defined:

(30) $$\begin{array}{c} \xi =\frac{1}{2}j\phi t,\;\;\nu =\frac{\kappa }{c_{R}}t,\\ \Phi =\sqrt{\nu^{2}+\xi^{2}},\;\;\Psi =\frac{\xi^{2}}{\nu^{2}}. \end{array}$$

**Figure 5:**
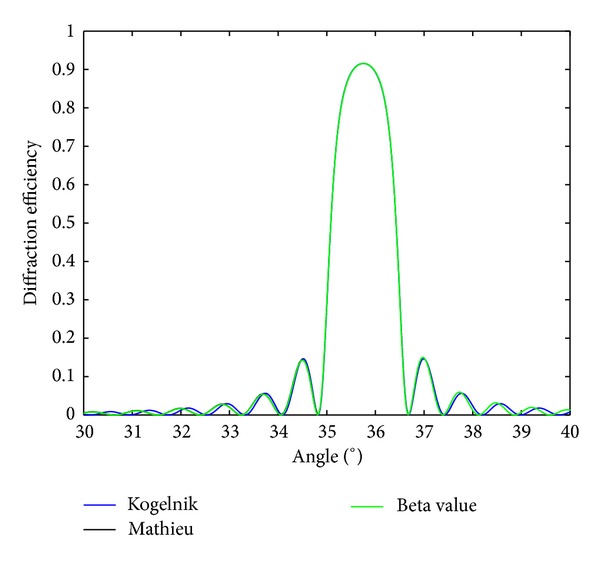
Diffraction efficiency of a reflection grating index-matched with thickness of 26 **μ**m, spatial frequency of 3800 lines/mm, average refractive index *n*_II_ = 1.5, and refractive index modulation of 0.012.

**Figure 6:**
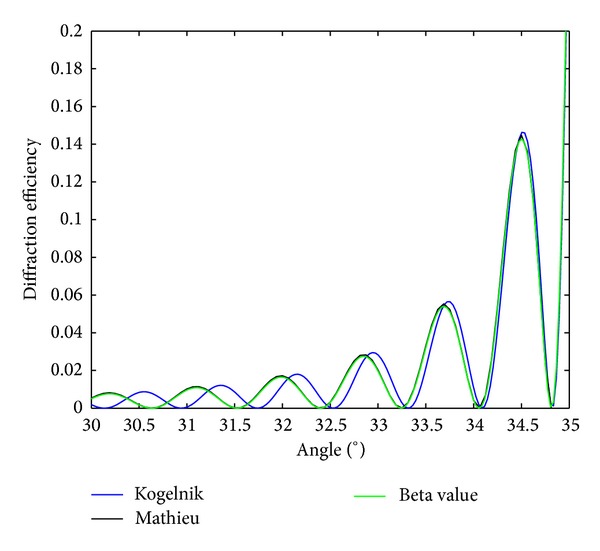
Diffraction efficiency of a reflection grating index-matched with thickness of 26 **μ**m, spatial frequency of 3800 lines/mm, average refractive index *n*_II_ = 1.5, and refractive index modulation of 0.012.

In Figure 5, the diffraction efficiency as a function of the angle is represented for the beta value and Kogelnik's models and also for the solution given in terms of Mathieu functions. The diffraction grating of the simulation has the following parameters: *t* = 26 **μ**m, spatial frequency of 3800 lines/mm, average refractive index *n*_II_ = 1.5, and refractive index modulation of 0.012. As can be seen near the Bragg angle, the three theories reproduce the same results. Nonetheless, a better look to the figure demonstrates that for out-of Bragg incidence the results obtained by using Kogelnik's theory deviate from the correct ones. This can be seen clearer in Figure 6 where a different angle range has been used to prove this matter. This demonstrates that even for index-matched media Kogelnik's theory is not appropriate to describe the efficiency of the diffracted order out of Bragg.

## 5. Partially Index-Matched Case

In this case, the reflection grating is embedded in two media, where the second is index-matched with the reflection grating.

In this case, *a* = 1, *b* = 1, *c* = 0, and *d* = 1; an explicit equation for the diffraction efficiency can be obtained:

(31) η=((a2−b2)ν2−2b2ξ2+2jabνξ  −[(a2+b2)ν2+2jabνξ]cos⁡⁡(2Φ)) ×((b2−a2)ν2−2a2ξ2+2jabνξ   −[(a2+b2)ν2+2jabνξ]cos⁡(2Φ))−1.

**Figure 7:**
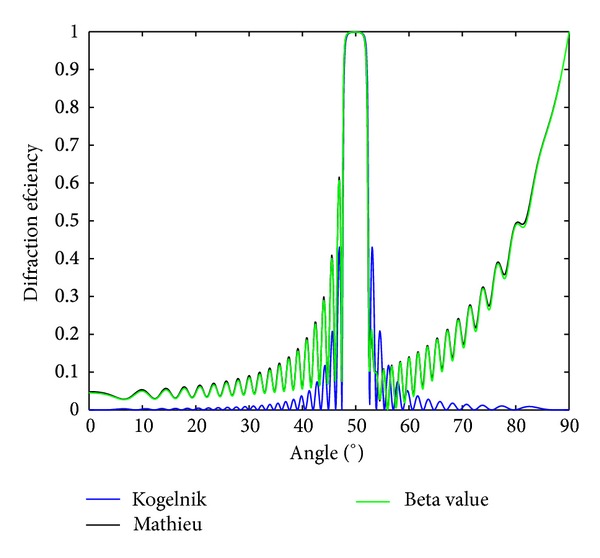
Diffraction efficiency of a reflection grating partially index-matched with thickness of 27 **μ**m, spatial frequency of 3700 lines/mm, average refractive index *n*_II_ = 1.4, and refractive index modulation of 0.025.

In Figure 7, the diffraction efficiency as a function of the angle is represented for the beta value and Kogelnik's models and also for the solution given in terms of Mathieu functions. The diffraction grating of the simulation has the following parameters: *t* = 27 *μ*m, spatial frequency of 3700 lines/mm, average refractive index *n*_2_ = 1.4, and refractive index modulation of 0.025 (slightly overmodulated). Again, as expected, a clear deviation of Kogelnik's theory is observed out of Bragg, but the size and position of the lobe corresponding to Bragg angle are well reproduced in this case.

## 6. Conclusions

In this work, we have described a beta value coupled wave model to study nonslanted reflection gratings. The main contribution of this work is the introduction of appropriate boundary conditions to solve the coupled differential equations. The results obtained using this method were compared with Kogelnik's coupled wave theory and a matrix method which gives the solutions in terms of Mathieu functions. In the case of index-matched media, the deviation of Kogelnik's model with respect to the one proposed and the matrix method for incidence off-Bragg has been demonstrated. For the case of a reflection grating embedded in air, it is clear that Kogelnik's theory no longer holds, but the proposed method gives accurate results.
